# Pharmacodynamics of cisatracurium in the intensive care unit: an observational study

**DOI:** 10.1186/2110-5820-4-3

**Published:** 2014-02-11

**Authors:** Eric Dieye, Vincent Minville, Karim Asehnoune, Claude Conil, Bernard Georges, Pierre Cougot, Olivier Fourcade, Jean-Marie Conil

**Affiliations:** 1 Department of Anesthesiology and Intensive Care Medicine, Faculté de Médecine Toulouse-Rangueil, EA 4564-MATN, Institut Louis Bugnard (IFR 150), CHU Toulouse, Université Toulouse III Paul Sabatier, F-31000 Toulouse, France; 2 Department of Anesthesiology and Intensive Care Medicine, CHU Nantes, Nantes, France

**Keywords:** Cisatracurium, ICU, Current anesthetic practice, Neuromuscular monitoring

## Abstract

**Background:**

Data from previous studies indicate that optimal conditions for intubation are met 120 seconds after administration of 0.15 mg.kg^-1^ cisatracurium (ED95 × 3) following the induction of anesthesia. The aim of this study was to compare the doses required for complete paralysis after induction of anesthesia in ICU patients with the dose used in patients undergoing elective surgery.

**Methods:**

Seventeen ICU patients undergoing percutaneous tracheostomy and 17 patients undergoing an elective surgical procedure under muscle relaxation were included. In both groups, an initial intravenous bolus of cisatracurium besylate was given at a dose of 0.15 mg.kg^-1^ followed by repeated boluses of 0.03 mg.kg^-1^ every four minutes. The objective was to obtain no response to the train-of-four (TOF). The contractile response of the corrugator supercilii muscle was monitored every minute by observing the TOF in response to a peripheral nerve stimulator with a constant current set to 60 mA.

**Results:**

After the initial dose of cisatracurium, none of ICU patients (0/17) versus 15/17 of the elective surgery patients were completely paralyzed (*P* < 0.0001). There was a delay in the onset of neuromuscular blockade among the ICU patients. The cumulative doses of cisatracurium were significantly higher in the ICU group with 38 ± 14 mg (that is, 10 ± 4.7 ED95) versus 11 ± 2 mg (that is, 3 ± 0.3 ED95) in the elective surgery group (*P* < 0.0001).

**Conclusion:**

The dosing of cisatracrurium for ICU patients, which is based on the dose recommended for elective anesthesia, is unsuitable because the onset is too slow. This phenomenon is probably caused by changes in the pharmacodynamics and pharmacokinetics. These data suggest that neuromuscular monitoring should be used in the ICU.

## Background

Although the use of neuromuscular blocking agents in the ICU is still a matter of ongoing debate, they continue to be frequently used. For example, in patients under volume controlled ventilation, a transient curarization can be used for brief diagnostic or therapeutic procedures in order to avoid the hemodynamic consequences of deep sedation. Also, in acute respiratory distress syndrome (ARDS) patients, a prolonged curarization of 48 hours or more is beneficial regarding systemic oxygenation, even in patients well-adapted to their ventilator [1]. Because of its pharmacokinetic properties, cisatracurium, a non-depolarizing neuromuscular blocking agent of the benzylisoquinoline family, is a popular choice in the ICU since it is (at physiological pH and temperature) rapidly degraded by Hoffmann elimination, yielding two metabolites without muscle relaxant properties: laudanosine and quaternary monoacrylate. Impaired renal or liver function has little influence on the pharmacokinetics of this drug [2,3]. The volume of distribution of cisatracurium is limited to the extracellular fluid (144 mL.kg^-1^), total plasma clearance is approximately 5.3 mL.min^-1^.kg^-1^, and the elimination half life is between 22 and 30 minutes [4,5].

In both the ICU and during anesthesia, the train-of-four (TOF) is the most widely used method of monitoring neuromuscular function [6-11]. The ED95 of cisatracurium is the dose required to produce a 95% twitch height depression for the first twitch response of the adductor pollicis muscle after stimulation of the ulnar nerve, and is approximately 0.05 mg.kg^-1^ of body weight during anesthesia with hypnotics and opioids (thiopental/midazolam/fentanyl) [12,13]. Optimal conditions for intubation are met 120 seconds after the administration of 0.15 mg.kg^-1^ cisatracurium (3 × ED95) during induction of anesthesia (for 89 to 100% of patients) [14,15]. After administration of 0.20 mg.kg^-1^ cisatracurium (4 × ED95), optimal conditions are met within 90 seconds (for 95 to 100% of patients) [15]. However, all the above studies were done on patients scheduled for elective surgery and thus these recommendations may not be appropriate for ICU patients where a variety of factors can affect the pharmacokinetics and pharmacodynamics of muscle relaxants: liver and/or kidney failure; hypothermia; fluid and electrolyte imbalances [16,17]; disorders of acid–base balance; as well as potential drug interactions (for example, aminoglycosides, steroids) [18]. This could indeed explain the observation that in clinical practice, higher doses of cisatracurium are often needed for ICU patients to obtain a clinically acceptable degree of neuromuscular blockade (TOF = 0).

The aim of this study was to compare the doses required for complete paralysis after induction of anesthesia in ICU patients with the dose used in patients undergoing elective surgery.

## Methods

Thirty-four patients were enrolled in this prospective observational study. The study was performed in accordance with the Helsinki Declaration. The Local Ethics Committee (comité d’éthique de la recherche des Hôpitaux de Toulouse: registration number 23–0412) approved the study. Because there was neither a randomization involved nor a change in the current local clinical practice (TOF monitoring is part of the routine medical care in the ICU and for surgical patients requiring a muscle relaxant), the institutional review board waived the requirement for a written informed consent.

Seventeen consecutive ICU patients who were paralyzed for a percutaneous tracheostomy were included. The indication for the tracheostomy was an anticipated length of mechanical ventilation for more than 15 days. ICU patients were stable (no acute hepatic or renal failure), euvolemic and on low dose catecholamine infusion (norepinephrine < 1 mg.h^-1^).

The comparison group consisted of 17 consecutive patients who were paralyzed for the anesthesia as part of an elective surgical intervention. Exclusion criteria included pregnancy, a history of significant neuromuscular disease (multiple sclerosis; myasthenia gravis; muscular dystrophies), a known allergy to a non-depolarizing neuromuscular blocking agent, patients who received neuromuscular blocking agents or were taking a treatment known to influence the neuromuscular junction (anticonvulsants; antidepressants; antihistamines). For each patient, demographics and biological data were recorded. The creatinine clearance (CrCl) was assessed by the simplified formula of the Modification of Diet in Renal Disease (sMDRD) [19].

In all patients, (elective surgical patients as well as the ICU patients) general anesthesia was induced with propofol (2.5 to 3 mg.kg^-1^) and sufentanil (0.1 to 0.2 mg.kg^-1^) and an initial bolus of cisatracurium at a dose of 0.15 mg.kg^-1^. This was followed by a propofol infusion and repeated injections of 0.03 mg.kg^-1^ cisatracrurium every four minutes until complete muscle paralysis (TOF = 0) was achieved [20]. Monitoring of neuromuscular blockade was performed using a peripheral nerve stimulator with constant current using self-adhesive skin electrodes placed at the temporal region. A current of 60 mA (rectangular stimulation, pulse duration of 200 or 300 microseconds) was administered four times (TOF) at a frequency of 2 Hz. An independent observer monitored the TOF semi-quantitatively by observing the contractile response of the corrugator supercilii every minute. The criterion for muscle relaxation was a complete absence of any twitch response. Any visible contraction was considered incomplete relaxation.

### Statistical analysis

Based on a pilot study involving ten anesthetic patients and ten ICU patients, we estimated that the difference in dose to obtain adequate curarization was about 60%. We calculated the number needed to find a difference of 60% in the dose of cisatracurium (in mg) with an alpha of 5% and a power (1-beta) of 90% to be 16 patients in each group.

Statistical analysis was performed using the software StatView® (SAS Institute Inc., version 5.0, Cary, NC, USA). Quantitative variables are presented as median (minimum-maximum) and qualitative data are given as number and percentage. Normal distribution of data was tested via the Kolmogorov-Smirnov test. As data were not normally distributed, groups were compared by the Mann–Whitney *U*-test for continuous variables and by Fisher’s exact test for categorical variables.

## Results

Demographic and biological data are presented in Table 1. The reasons for ICU admission were: severe trauma (4); septic shock (1); acute respiratory failure (7); cerebral toxoplasmosis (1); aortic dissection (1); severe aortic stenosis (1); severe hyponatremia (1); and severe hypoglycemic coma (1). The ICU patients had been ventilated for 12 ± 5 days before they were included in the present study. No adverse cardiovascular events or signs of histamine release were observed in the two groups after administration of cisatracurium.

**Table 1:** Demographic and biologic data

	**ICU patients**	**Surgical patients**	***P***
	**N = 17**	**N = 17**	
**Age (years)**	60 (28 to 83)	56 (26 to 86)	0.89
**Sex (M/F)**	13/4	11/6	0.88
**Weight** (kg)	75 (55 to 128)	71 (51 to 83)	0.076
**Height** (cm)	160 (144 to 175)	169 (158 to 186)	0.099
**BMI**	29 (27 to 39)	26 (22 to 37)	0.083
**Urea** (mmol.L^-1^)	10.9 (3.60 to 31)	6 (3.4 to 23.6)	0.02^a^
**Creatinine** (μmol.L^-1^)	84 (41 to 211)	88.50 (63.6 to 407)	0.67
**Creatinine clearance**	86.7 (29.8 to 159.4)	76.6 (13 to 113.4)	0.42
(ml.min^-1^.1.73 m^2-1^ )			
**Prothrombin rate**	66 (20 to 89)	87 (64 to100)	0.001^a^
**SGOT (**IU.L^-1^**)**	40 (14 to 274)	34 (14 to 183)	0.33
**SGPT (**IU.L^-1^**)**	44 (15 to 425)	57 (13 to 293)	0.52
**Protein** (g.L^-1^)	55 (35 to 65)	71 (60 to 80)	< 0.001^a^

Data are presented as median (minimum - maximum); ^a^*P* < 0.05 using the Mann–Whitney *U*-test.

After the initial dose of cisatracurium, none of ICU patients (0/17) versus 15/17 anesthesia patients were completely paralyzed (*P* < 0.0001).

**Figure 1:**
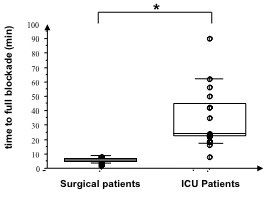
**Time of neuromuscular blockade for ICU (1) and surgical patients (0).** * = *P* < 0.05 using the Mann–Whitney *U*-test.

**Figure 2:**
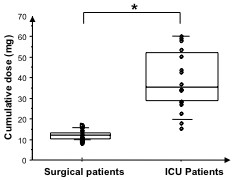
**Cumulative doses of cisatracurium for ICU (1) and surgical patients (0).** * = *P* < 0.05 using the Mann–Whitney *U*-test.

The time to achieve complete neuromuscular blockade (after the initial dose of 0.15 mg.kg^-1^ followed by repeated injections of 0.03 mg.kg^-1^ every four minutes) was much longer in ICU patients when compared to surgical patients: 34 ± 21 versus 4 ± 2 minutes respectively (*P* < 0.0001; Figure 1) and the total cumulative dose of cisatracurium was significantly higher in ICU patients when compared to surgical patients: 38 ± 14 mg versus 11 ± 2 mg respectively (*P* < 0.0001; Figure 2).

## Discussion

This is the first study comparing the dose of muscle relaxant needed between ICU patients and surgical patients. We found that the time to achieve a complete neuromuscular block after an initial dose of 0.15 mg.kg^-1^ followed by additional smaller repeat doses was longer in ICU patients when compared to surgical patients. We also showed that the cumulative doses of cisatracurium required to obtain a TOF = 0 were significantly higher in ICU patients.

The results observed in our surgical patients are consistent with other published studies [12-14]. Increasing doses of cisatracurium can shorten the time to obtain a TOF = 0 [12-14]. ICU studies regarding the pharmacokinetics and pharmacodynamics of cisatracurium focused on patients receiving continuous infusions of cisatracurium [1] and mostly described the recovery profiles after continuous administration [21,22]. The doses used in those publications were a bolus of 0.1 mg.kg^-1^ (2 × ED95) with an average rate of infusion of 2.6 mg.kg^-1^.min^-1^ (0.15 mg.kg^-1^.h^-1^) or 3.2 mg.kg^-1^.min^-1^ (0.19 mg.kg^-1^.h^-1^) as required to obtain a sufficient degree of neuromuscular blockade for mechanical ventilation [21,22]. In comparison, the maintenance of muscle relaxation during anesthesia for a 95% depression of muscle contraction has been shown to range between 1.4 mg.kg^-1^.min^-1^ (0.08 mg.kg^-1^.h^-1^) [12] and 1.5 mg.kg^-1^.min^-1^ (0.09 mg.kg^-1^.h^-1^) [23]. This suggests that the rate of continuous infusion required to obtain muscle relaxation is greater in ICU patients when compared to surgical patients.

The evaluation of cumulative dosing of neuromuscular blocking agents is one method to determine dose–response studies; however this method has limitations because the elimination process begins while subsequent doses of cisatracurium would continue to be administered and therefore recovery of neuromuscular blockade occurs during re-administration of cisatracurium. No study has so far compared the time required for muscle relaxation or the doses required to reach a maximum block after administration of boluses of cisatracurium in ICU patients when compared to surgical patients, but our results seem to be consistent with those described for the continuous infusion of muscle relaxants. In fact, the onset time may have been shorter if ICU patients had received a higher initial bolus dose. Thereby, our study only shows that in ICU patients the initial dose 0.15 mg.kg^-1^ was not adequate.

In ICU patients, a change in pharmacokinetics of cisatracurium may explain slower onset of paralysis and the need to administer larger doses. Indeed, Boyd *et al*. showed that the volume of distribution (21.9 versus 9.2 L) and total clearance (549 versus 293 mL.min^-1^) of cisatracurium are higher in ICU patients when compared with anesthesia [21], when the half-life of the drug remained unchanged [21]. In ICU patients, an increased volume of distribution could lead to a decrease in the concentration at the neuromuscular junction after the first dose and this may therefore explain the need to administer larger doses. Another explanation could be a deregulation of acetylcholine receptors in ICU patients [24]. This would involve qualitative and quantitative changes of the acetylcholine receptor of the neuromuscular junction [25]. An increase in the number of acetylcholine receptors (up-regulation), a proliferation of the receptors at the neuromuscular junction and the rest of the muscle membrane, but also the formation of neoreceptors were observed [24,26]. Neoreceptors differ from the native receptor by substitution of the e-subunit by the g-subunit [25] and are more resistant to the action of nondepolarizing neuromuscular blocking [27]. The result is a decreased sensitivity to nondepolarizing neuromuscular blocking agents and a requirement for larger doses to observe the same effect. This effect could in part explain the findings in the present study since our ICU patients had already been admitted and ventilated for 12 ± 5 days before they were studied.

In ICU patients, many others factors could interfere with the effect of neuromuscular blocking agents, for example the presence of hypokalemia, hypercalcemia, hypothermia, treatment with magnesium sulfate, and so on. However, in our study, the ICU patients were considered stable so these factors were unlikely to have played a significant role. This should be taken into account for future studies.

Our study has several limitations. First, in the absence of pharmacokinetic data no definitive conclusion regarding the etiology for the difference between groups can be drawn from this report. Second, this was an observational and not a randomized study.

## Conclusion

In summary, the doses of cisatracurium for ICU patients should be higher than those used in current anesthetic practice in order to achieve an adequate degree of neuromuscular blockade. This phenomenon could be related to changes in pharmacodynamics (deregulation of receptors) and pharmacokinetics (increase in the volume of distribution). Our data suggest that neuromuscular monitoring should be used to ensure a satisfactory degree of neuromuscular blockade in ICU patients requiring deep neuromuscular relaxation (TOF = 0) for surgical procedures.

Further dose–response studies are required to determine the optimal loading dose of cisatracurium in ICU patients.

### Key messages

•Repeated boluses and more time are necessary to achieve complete muscle relaxation in ICU patients if using the same dose as for the routine induction of anesthesia.

•Doses of cisatracurium should be higher than those used in current anesthetic practice for an adequate degree of neuromuscular blockade.

•It seems reasonable to recommend monitoring neuromuscular blockade in ICU patients.

## Abbreviations

ARDS: Acute respiratory distress syndrome; CrCl: Creatinine clearance; sMDRD: Simplified Modification of Diet in Renal Disease; TOF: Train-of-four.

## Competing interests

The authors declare that they have no competing interests.

## Authors’ contributions

ED, CC and PC carried out the patients’ inclusions. KA helped to draft the manuscript and reviewed the intellectual content. BG and OF participated in the design of the study and helped to draft the manuscript. JMC and VM conceived of the study, and participated in its design and coordination, helped to draft the manuscript and performed the statistical analysis. All authors read and approved the final manuscript.
